# East Australian Current velocity, temperature and salinity data products

**DOI:** 10.1038/s41597-023-02857-x

**Published:** 2024-01-02

**Authors:** Bernadette M. Sloyan, Rebecca Cowley, Christopher C. Chapman

**Affiliations:** CSIRO Environment, Hobart, 7004 Australia

**Keywords:** Physical oceanography, Physical oceanography

## Abstract

The East Australian Current (EAC) is the complex, highly energetic western boundary current that flows along the east coast of Australia. The EAC and its associated turbulent eddies dominate the marine climate of the Coral and Tasman Seas and the eastern Australian continental shelf. Here we present a series of consistent EAC data products that combines *in situ* temperature, salinity and velocity observations from the Australian Integrated Marine Observing System (IMOS) and Commonwealth Scientific and Industrial Research Organisation (CSIRO) EAC mooring array and North Stradbroke Island reference site. The EAC data products are produced from over 1000 individual instrument files collected during six 18-month deployments in the East Australian Current (EAC) off Brisbane, Australia at 27 °S between 2012–2022. The mooring individual instrument files are combined, interpolated onto a common vertical, temporal (hourly and daily) and spatial grid. The ITCOMPSOM method is used to fill missing data in the time series. These data product can be used to investigate intra- and interannual EAC variability and boundary current dynamics.

## Background & Summary

**Fig. 1:**
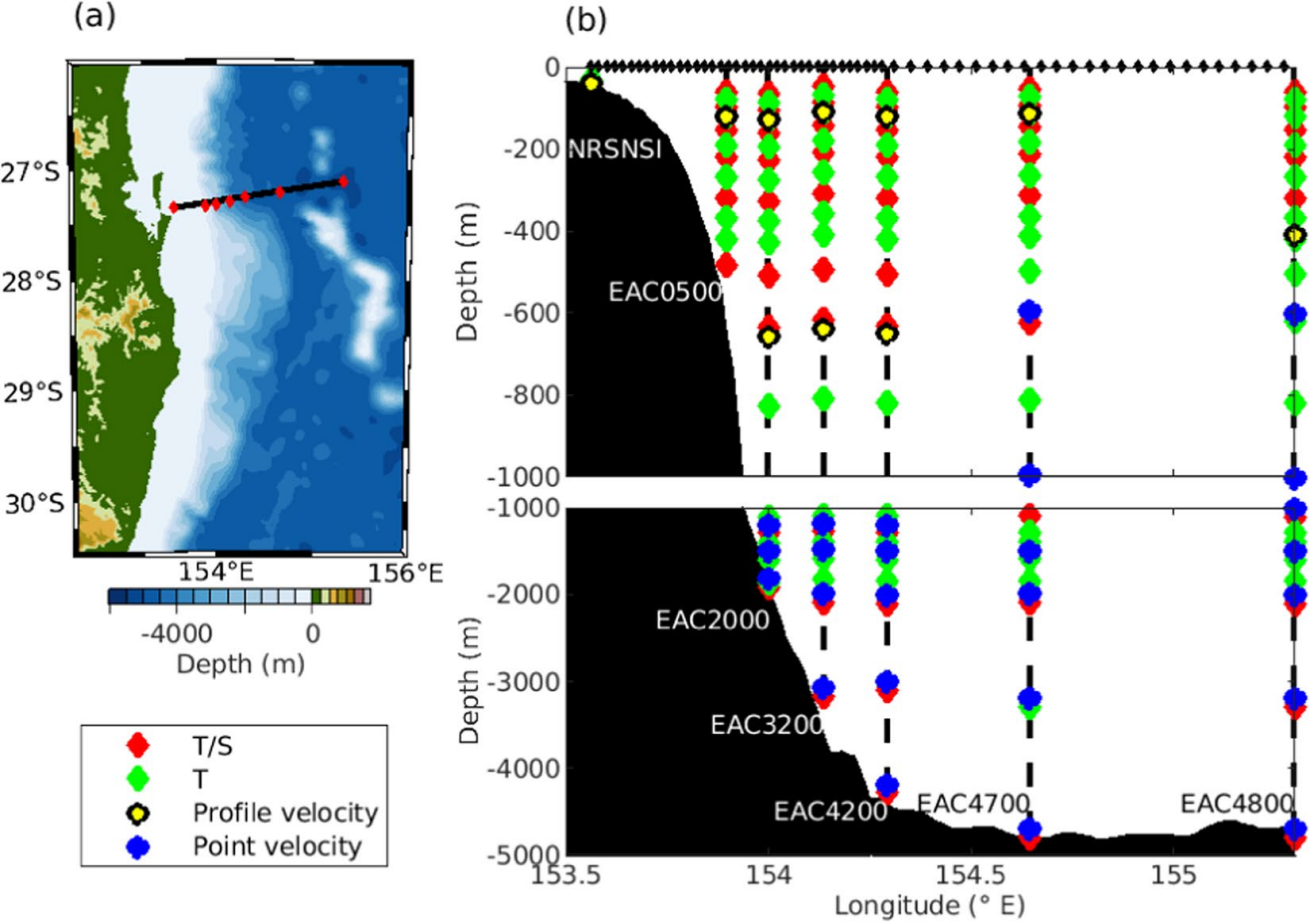
(**a**) Location of the EAC moorings (red diamond) and the regular spatial grid the mooring data are interpolated onto (black line). (**b**) Vertical distribution of instruments at each mooring (as deployed from April 2018 and June 2022). The moorings are from the continental shelf to the off-shore deep ocean: National Reference Station North Stradbroke Island (NRSNSI), East Australian Current moorings at nominal depths of 500 m (EAC0500), 2000 m (EAC2000), 3200 m (EAC3200), 4200 m (EAC4200), 4700 m (EAC4700) and 4800 m (EAC4800). Instruments and variables observed are Temperature (T) and salinity (S) red diamond, temperature (T) green diamond, velocity profiles of 150 m, 300 m, and 600 m length yellow circle and single point velocity blue circle are shown. Black diamonds show every 2nd grid point that the mooring velocity, temperature and salinity are mapped to.

The East Australian Current (EAC) is the complex, highly energetic western boundary current (WBC) of the South Pacific Ocean that flows along the east coast of Australia. As the strongest ocean current in the region, the EAC is responsible for moving vast amounts of water, heat and other properties south from the tropics to the temperate latitudes which impacts the weather, ocean environment and, composition and functioning of marine ecosystems along the entire east coast of Australia^1–4^.To characterise the EAC’s intra- and inter-annual variability, the Australian Integrated Marine Observing System (IMOS) and CSIRO supported a comprehensive *in situ* mooring array at approximately 27 °S (Fig. 1), collecting full-depth observations of velocity, temperature and salinity between 2012 and 2022 when funding of the mooring array ceased (Table 1). The mooring array provided an unprecedented time-series of *in situ* direct velocity, temperature, and salinity observations at the seven-mooring sites across the entire EAC extending from the continental shelf to the deep abyssal basin.

**Table 1:** Information on the mooring deployments used to create the vertically, spatially and daily interpolated gridded data product.

Mooring ID	NRSNSI	EAC0500	EAC2000	EAC3200	EAC4200	EAC4700	EAC4800
Water Depth (m)	63	545	1905	3185	4267	4779	4791
Latitude (°N)	−27°20.5	−27°19.6	−27°18.9	−27°17.1	−27°15.0	−27°12.5	−27°06.3
Longitude (°E)	153°33.7	153°54.0	153°59.5	154°8.2	154°17.9	154°38.9	155°18.3
Apr 2012-Aug 2013	✓	✓*	✓		✓	✓	✓
May 2015-Oct 2016	✓	✓	✓	✓	✓	✓	✓
Oct 2016-Apr 2018	✓	✓	✓	✓	✓	✓	✓
Apr 2018-Sep 2019	✓	✓	✓	✓	✓	✓	✓
Sep 2019-May 2021	✓	✓	✓	✓	✓	✓	✓
May 2021-June 2022	✓	✓	✓	✓	✓	✓	✓

The mooring array was not deployed for a 22-month period in 2013–2015 between the decommissioning of RV Southern Surveyor and commissioning of the RV Investigator. * for April 2012 to August 2013 we use IMOS SEQ0400 mooring.

While ocean mooring arrays provide the most effective and efficient way to directly measure the temporal and spatial variability of ocean currents and properties simultaneously in the narrow WBCs^5^, the raw data as downloaded from the moored instruments are not readily usable for various reasons by non-experts. For example, mooring design improvements/modification are generally implemented during the life time of the array between consecutive deployments, including addition/removal of instruments or changes to nominal instrument deployment depths. Instruments have different sampling rates and they may fail during deployment. Additionally, the quality controlled (QC) and assured (QA) data procedures assess each instrument independently and flag instrument data as good, probably good, probably bad, bad or derived, resulting in very different QC/QA characteristics through time as different instruments types are deployed or moved throughout the mooring arrays deployment period. To further complicate matters, the depth of an instrument varies with time, as a result of the mooring structure being physically ‘blown-over’ by ocean currents. Thus, the resulting time-series data will have an irregular structure that varies amongst the moorings and deployments^6,7^. Further post-processing of the mooring data including filling vertical and temporal data gaps^8^, interpolating to common depth and temporal frequencies, and spatially between moorings are generally undertaken to produce mooring array data products that are readily usable by a variety of users.

Here we introduce a series of EAC mooring-based data products to enable wider use of this comprehensive time series data set (Table 2). The three data products are built consecutively starting with the East Australian Current individual mooring hourly and depth (vertical resolution 10 m from sea surface to 400 m, and 20 m resolution from 400 m to bottom) gridded data product (Table 3) that is available from 10.25919/7mkz-3928. This data product contains hourly- and depth-gridded products of currents, temperature and salinity for each mooring (Table 3) that are produced from the quality-controlled velocity, temperature and salinity instrument files for each EAC mooring over the six approximately 18-month deployments^6,7^ and the national reference station North Stradbroke Island mooring^9,10^ for the corresponding time period. The hourly velocity, temperature and salinity time-series contains missing value when an instrument either failed, was lost or data were flagged bad. From the hourly-depth gridded mooring data product, we produce the East Australian Current individual mooring daily and depth gridded data product (Table 4) that is available from 10.25919/10h0-yf37. For each mooring, the file that contains two variables for velocity, temperature and salinity (e.g. TEMP and TEMP_FILLED, Table 4). The temperature, salinity and velocity variables have data gaps (temporal and vertical). The temperature, salinity and velocity filled variables are continuous in time and depth, except for the 22-month period between September 2013 and April 2015 when the EAC mooring array was not deployed. Data gaps are filled using the Iterative Completion Self Organising Maps (ITCOMPSOM) method^8^. Finally, we spatially interpolate the mooring temperature, salinity and velocity filled timeseries data across the mooring array from the national reference station North Stradbroke Island mooring site to the EAC4800 mooring site to create the East Australian Current gridded (depth, distance and time) mooring product (Table 5) which is available from 10.25919/sfw7-hc46.

**Table 2:** List of EAC mooring data products.

Data Product	Citation	DOI
Individual mooring hourly and 10–20 m depth gridded	Sloyan and Cowley^12^	10.25919/yycf-ed65
Individual mooring daily and 10–20 m depth gridded	Sloyan, Cowley and Chapman^13^	10.25919/egsf-yh54
Gridded (depth, distance and time) mooring product	Sloyan, Cowley and Chapman^21^	10.25919/wg81-e165

**Table 3:** Variables included in individual mooring hourly and depth gridded netCDF file.

Parameter	Variable Name	Units
Time	TIME	days since 1950-01-01 00:00:00 UTC
Depth	DEPTH	meters (m)
Longitude	LONGITUDE	°N
Latitude	LATITUDE	°E
Temperature	TEMP	°C (ITS-90)
Temperature Uncertainty	TEMP_uncertainty	°C (ITS-90)
Salinity	PSAL	PSU (PSS-78)
Salinity Uncertainty	PSAL_uncertainty	PSU (PSS-78)
U velocity	UCUR	m/s^−1^ (true east)
U velocity Uncertainty	UCUR_uncertainty	m/s^−1^ (true east)
V velocity	VCUR	m/s^−1^ (true north)
V velocity Uncertainty	VCUR_uncertainty	m/s^−1^ (true north)

Netcdf variable dimension are time and depth.

**Table 4:** Variables included in individual mooring daily and depth gridded netCDF file.

Parameter	Variable Name	Units
Time	TIME	days since 1950-01-01 00:00:00 UTC
Depth	DEPTH	meters (m)
Longitude	LONGITUDE	°N
Latitude	LATITUDE	°E
Temperature	TEMP	°C (ITS-90)
Temperature Uncertainty	TEMP_uncertainty	°C (ITS-90)
Temperature Filled	TEMP_FILLED	°C (ITS-90)
Temperature Filled Uncertainty	TEMP_FILLED_uncertainty	°C (ITS-90)
Salinity	PSAL	PSU (PSS-78)
Salinity Uncertainty	PSAL_uncertainty	PSU (PSS-78)
Salinity Filled	PSAL_FILLED	PSU (PSS-78)
Salinity Filled Uncertainty	PSAL_FILLED_uncertainty	PSU (PSS-78)
U velocity	UCUR	m/s^−1^ (true east)
U velocity uncertainty	UCUR_uncertainty	m/s^−1^ (true east)
U velocity Filled	UCUR_FILLED	m/s^−1^ (true east)
U velocity Filled uncertainty	UCUR_FILLED_uncertainty	m/s^−1^ (true east)
V velocity	VCUR	m/s^−1^ (true north)
V velocity uncertainty	VCUR_uncertainty	m/s^−1^ (true north)
V velocity Filled	VCUR_FILLED	m/s^−1^ (true north)
V velocity Filled uncertainty	VCUR_FILLED_uncertainty	m/s^−1^ (true north)

Netcdf variable dimensions are time and depth.

**Table 5:** Variables included in mooring array daily, spatial and depth gridded netCDF file.

Parameter	Variable Name	Units
Time	TIME	days since 1950-01-01 00:00:00 UTC
Depth	DEPTH	meters (m)
Longitude	LONGITUDE	°N
Latitude	LATITUDE	°E
Bottom Topography	BOTTOM_DEPTH	meters (m) ETOPO1 01 arc-minute
Temperature	TEMP	°C (ITS-90)
Temperature Uncertainty	TEMP_uncertainty	°C (ITS-90)
Salinity	PSAL	PSU (PSS-78)
Salinity Uncertainty	PSAL_uncertainty	PSU (PSS-78)
U velocity	UCUR	m/s^−1^ (true east)
U velocity	UCUR_uncertainty	m/s^−1^ (true east)
V velocity	VCUR	m/s^−1^ (true north)
V velocity	VCUR_uncertainty	m/s^−1^ (true east)

Netcdf variable dimensions are time, depth and longitude. The filled variables of the individual mooring daily and depth gridded product (Table 4) are used to produce this data product.

The EAC mooring gridded products (Table 2) will support a large variety of analysis techniques and various research uses. The data products can be used to determine temporal and spatially variability of the EAC, current dynamics, ocean mixing, property transport and variability and much more. These data products will enable comparison to and combination with other ocean observation platforms and, ocean and earth system models. Research undertaken using these data products will provide improved insights into the variability of the EAC and combined with other observational and model data lead to a more complete understanding of this important current and western boundary current dynamics.

## Methods

### Data products

The EAC mooring-based data products are derived from velocity, temperature and salinity observations from the EAC mooring array and the national reference station North Stradbroke Island (NRSNSI) mooring (Fig. 1 and Table 1). Direct velocity observations are provided by acoustic doppler current profiling (ADCP) instruments of various frequencies (75, 150, and 300 kHz), and point source ADCP velocity instruments. The profiling ADCPs provided vertical velocity profiles over the full water depth for moorings in water depths shallower than 500 m (NRSNSI and EAC0500) or upper 1000 m for mooring on the continental slope (EAC2000, EAC3200 and EAC4200) at varying vertical resolutions (4 m, 8 m, and 16 m). At the off-shore moorings (EAC4700 and EAC4800), ADCPs provide vertical velocity profiles (16 m resolution) over the upper 600 m and 500 m, respectively. Point source velocity data, below the ADCP profiling depth, provide observations to the sea floor at vertical resolutions of between 500 m to 1000 m. The moorings provided temperature and salinity observations from approximately 20 m below the surface to the sea floor at varying vertical resolution. Temperature observations were obtained from Sea-Bird Electronic (SBE) instruments (SBE39-plus and SBE37-SMP MicroCAT) and, on early mooring deployments, Starmon Mini Star Oddi instruments. Salinity observations were obtained from SBE 37-SMP MicroCAT instruments. Temperature vertical resolution is 20 m between 20 to 200 m, 50 m between 200 to 400 m, 100 m between 400 to 800 m, and 200 m to 300 m between 800 m and the seafloor. Salinity observations are resolved at a vertical resolutions of 50 to 200 m between 20 m to 1000 m and 500 m to 1000 m between 1000 m and the seafloor. The temperature and salinity vertical resolutions are similar for all moorings. Note that the vertical resolution of salinity observations in the upper 1000 m were improved with every subsequent mooring deployment.

The 2012–2013 EAC mooring array consisted of seven moorings that extended from a water depth of 200 m on the continental slope to approximately 4800 m water depth on the abyssal plain^11^. In 2015, after a 22-month hiatus, a redesigned EAC six mooring array was deployed (Fig. 1). As the 2012–2013 and 2015–2022 EAC mooring array designs are not exactly the same, the moorings are combined as follows. The 2012–2013 moorings at 200 m (SEQ0200) and 1500 m (EAC_M1) are not used in this study. Since 2015 a mooring at 500 m (EAC0500) on the continental slope was maintained. The EAC0500m mooring replaced a previous IMOS Southeast Queensland mooring at 400 m (SEQ0400) that was deployed between 2012–2013. Given that the distance separating the EAC0500 and SEQ0400 moorings was only 2.47 km, we have combine these two moorings to provide upper continental slope observations for the 2012–2022 time period. The NRSNSI mooring site, in 63 m water depth, was continuously occupied during the period of EAC mooring array. Here we combine the 2012–2013 and 2015–2021 EAC mooring arrays with the North Stradbroke Island mooring to build a successive series of EAC mooring-based data products. All EAC products have a 22-month data gap between 2013 and 2015 when the EAC mooring array was not deployed.

The hourly-depth individual mooring gridded data^12^ are created from the observed data contained in IMOS FV01* mooring instrument temperature and salinity files^6,10^ and current files^7,9^. The deployment FV01* files contain quality controlled data and flags for each instrument. The individual instrument files are screened to find data flagged as ‘good or changed’ (flags 1, 2 and 5) and arranged in consecutive time and increasing depth. Surface reflection errors contaminate the near-surface upper 2 ADCP observation bins, thus our shallowest ocean velocity data is at 20 m below the surface. We estimate salinity data for mooring instruments that only provide observations of temperature by determining a temperature-salinity (T-S) relationship at each mooring site from the coincident temperature and salinity mooring observations and mooring voyage ship-based Conductivity-Temperature-Depth (CTD) profiles. A 10th-order polynomial was used to uniquely determine salinity from the T-S data. Using the polynomial fit, salinity estimates for instruments below 100 m that measured temperature only were derived. Temperature, salinity and velocity data are then linearly interpolated onto a common time grid (hourly) and common depth grid (10 m to 400 m depth and 20 m from 420 m depth to the bottom) for each deployment. Periods where an instrument fails or data is missing are filled with missing values. Deployments are then concatenated together to give a hourly-depth temperature (TEMP), salinity (PSAL) and velocity (UCUR, VCUR) dataset for each mooring site.

From the hourly-depth individual EAC mooring data product^12^ we produce the daily-depth gridded EAC mooring data product^13^. A 5-day filter was applied to the hourly-depth mooring data to remove tides and other high frequency processes. All data were then interpolated to a common daily time stamp to create the daily-depth velocity (UCUR and VCUR) data and temperature (TEMP) and salinity (PSAL) data. We then apply an Iterative Completion Self-Organizing Maps (ITCOMPSOM) method^8^, based on the SOM (Self Organising Maps) neural network machine learning algorithm^14–16^, to fill temporal and vertical data gaps and create the time and depth continuous velocity (UCUR_filled, VCUR_filled), temperature (TEMP_filled) and salinity (PSAL_filled) variables. ITCOMPSOM inputs missing values in the daily data vector several times from progressively larger topological maps that combine previously completed data with new data with missing values at each iteration (see Sloyan *et al*. 2023 their Fig. 5).

The EAC moorings do not have a surface buoy as they are in a busy shipping and fishing region and the shallowest temperature or salinity observation is 20 m below the surface or deeper. We use the NOAA/NESDIS/NCEI Daily Optimum Interpolation Sea Surface Temperature (OISST), version 2.1 data^17^ (https://www.ncdc.noaa.gov/oisst/optimum-interpolation-sea-surface-temperature-oisst-v21) and OCEAN/IPSL sea surface salinity (SSS) L3 version 5 data from SMOS (Soil Moisture and Ocean Salinity) satellite^18,19^ (https://www.seanoe.org/data/00417/52804/) to provide contemporaous surface temperature and salinity data. We linearly interpolate the OISST and SMOS data to the mooring location and daily time stamp. The OISST and SSS data are added to the mooring temperature and salinity data at 0 m prior to application of the ITCOMPSOM method. Sloyan *et al*.^8^ provide details on the ITCOMPSOM method as applied to the EAC mooring array data. Following Sloyan *et al*.^8^ all the mooring velocity data are arranged into a velocity data matrix and the mooring temperature and salinity data are used to construct a combined temperature and salinity data matrix. The time period between September 2013 and April 2015 when no EAC mooring data is available is removed and the matrices are sorted by the total number of data per day from greatest number of data to least data. Ancillary information added to the ITCOMPSOM velocity and temperature and salinity data matrices includes day of year to incorporate seasonality information, and distance from a geographical point just inshore of the NRSNRS mooring location to provide EAC jet coherence information. In addition, we interpolate the daily IMOS sea surface height anomaly to the mooring locations and append this to the velocity data matrix. All data matrices are normalised by their property variance. We build the SOM used to fill the missing velocity and, temperature and salinity data by iterating the velocity and, temperature and salinity matrices over 25 iterations while progressively increasing the number of SOM neutral classes from an initial number of 200 for velocity and 100 for temperature and salinity, respectively, to a final number of classes of 1300 for velocity and 550 for temperature and salinity. We used the Davies-Bouldin index^20^ to determine the minimum number of neutral classes. At the completion of the iteration loop the matrices are denormalised and reordered to sequential time, and the velocity, temperature and salinity data for each mooring provide the mooring filled data set. Finally we assume a constant velocity from 20 m to the sea surface.

The final EAC mooring-based data product is the depth, distance and time gridded time-series of velocity, temperature and salinity^21^. This product is created by constructing a 2-dimensional distance (longitude)–depth regular grid using bathymetry from ETOPO1^22,23^ along the mooring line (Fig. 1), then interpolating the filled mooring velocity, temperature and salinity^13^ data between the inshore (NRSNSI) and most offshore (EAC4800) mooring onto this regular grid. The product is available from 10.25919/sfw7-hc46. Across the Australian shelf and continental slope, where the topographic slope is steep and average distance between moorings is 0(10 km), the grid resolution is 1 km (NRSNSI to EAC4200). Over the abyssal plain, from the base of the continental slope (EAC4200) to the outer mooring (EAC4800), where the distance between mooring is 35 km and 65 km, a 2 km resolution is used. For velocity we apply a no-slip bottom boundary condition at the deepest grid cell and assume that the flow is 0 m s^−1^ at the bottom. For temperature and salinity, the bottom boundary condition is the deepest filled-temperature and salinity value, respectively. We apply a natural-neighbor interpolation^24^ to interpolate between the boundary conditions and the mooring sites to the distance(longitude)-depth grid at a daily time-step. The natural-neighbor interpolation method has a number of advantages over other interpolation methods (e.g. linear or nearest-neighbor), including continuity and that it is spatially adaptive.

### Data uncertainty estimates

Uncertainty estimates are included for the temperature, salinity and velocity variables for each EAC data product. The estimated uncertainties start from the expanded uncertainties obtained from the instrument calibration certificates. Uncertainty estimates are propagated through each analysis step to obtain time (hourly and daily), depth and spatially independent uncertainty estimates for all variables.

SBE37, SBE39 and Starmon Mini Star Oddi instrument uncertainties are an average of expanded uncertainties taken from CSIRO calibration certificates. Salinity uncertainty for SBE37 instruments is calculated from the calibration certificate conductivity uncertainties using the TEOS-10^25^ as:

$${\rm{Salinity}}\;{\rm{uncertainty}}={\rm{gsw}}\_{\rm{SP}}\_{\rm{from}}\_{\rm{C}}\left(C1-C2,{T}_{0},{P}_{0}\right)-{\rm{gsw}}\_{\rm{SP}}\_{\rm{from}}\_{\rm{C}}\left(C2,{T}_{0},{P}_{0}\right)$$

where conductivity C1 = 42.918 mS/cm, C2 = 42.916 mS/cm, Temperature (T_0_) = 15 °C and Pressure (P_0_) = 10.1325 dbar. We have assumed that the SBE37 pressure uncertainty is small and therefore do not include a pressure uncertainty in the data products or the influence of pressure uncertainty on estimated temperature and conductivity. For derived salinity, based on the T-S relationship, the uncertainty estimate is the root-mean-square error (RMSE) of the known salinity (observed mooring salinity and ship CTD data) and the derived salinity from the polynomial fit. Thus the uncertainties of the salinity data in the hourly-depth individual mooring data^12^ are either the expanded uncertainty from the calibration certificates or RMSE error of the T-S fit (Table 6). For single point velocity observations we use the Nortek Aquadopp uncertainties from the instrument setup files and instrumental uncertainty from the manufacturer^26^ (Table 6). Teledyne RDI ADCP uncertainties for current profiles are calculated from the standard deviation of error velocity^27^ for each velocity bin calculated using the RDI processing software (Table 6). These uncertainties are combined and provide uncertainty estimates for the observed hourly and daily gridded products.

**Table 6:** Variable uncertainty estimates derived from the calibration certificates for SBE39 and SBE37, and Nortek instruments.

	PSAL_uncertainty (PSU) (PSU)	TEMP_uncertainty (°*C*)	UCUR_uncertainty (m s^−1^)	VCUR_uncertainty (m s^−1^)
SBE 37	0.0018	0.0015		
SBE39		0.0015		
Derived Salinity				
NRSI, EAC0500	0.0527			
EAC2000	0.0444			
EAC3200	0.0410			
EAC4200	0.0382			
EAC4700	0.0384			
EAC4800	0.0402			
RDI velocity			0.0010–0.0370	0.0010–0.1000
Nortek velocity			0.009	0.009
SOM	0.0364	0.3848	0.0299	0.0601
Spatial mapping	0.02 - 0.089	0.01 - 0.394	0.01–0.047	0.01–0.099

Inferred salinity uncertainties are the RMSE of the difference between observed and derived salinity for each mooring. RDI velocity uncertainty range obtained from the processing software is given. SOM uncertainty is RMSE difference between known and predicted data of 20% random withholding of known data from 100 SOM realisations. The spatial mapping uncertainty range from difference of perturbed mapping is provided.

To estimate the uncertainty associated with the use of the ITCOMPSOM method to create filled missing data, we preformed a monte-carlo boot strapping procedure using 100 SOM realisations withholidng 20% of known velocity, temperature and salinity data^8^. The SOM filled data uncertainties are then calculated as the RMSE difference between known data and predicted data (Table 6). The filled data uncertainties are derived as a combination of the instrument extended uncertainty, velocity profile uncertainties, the salinity derived uncertainty and SOM uncertainty. Finally, we estimate the spatial mapping uncertainty as the difference in mapped products that are perturbed by the velocity, temperature and salinity filled data uncertainty matrices (Table 6).

## Data Records

The EAC gridded products (Table 2) are published at the CSIRO Data Portal (10.25919/7mkz-3928, 10.25919/10h0-yf37 and 10.25919/sfw7-hc46). The format is a netCDF data file for each individual mooring for the hourly-depth^12^ and daily-depth^13^ data products. The dimensions of the variables are time (hourly, daily) and depth (m) for the velocity, temperature and salinity data (Tables 3, 4). The EAC mooring-based daily, spatial and depth gridded product^21^ is a single file netCDF file with dimension of time (daily), depth (m) and longitude (°N) (Table 5). IMOS netCDF file conventions^28^ are used for all files.

## Technical Validation

The EAC mooring-based data products are built from the quality controlled data flagged as ‘good or changed’ (flags 1, 2 and 5)^29–34^. A polynomial fit determined from temperature and salinity data from mooring SBE37SMP instrument and ship-based CTD profiles was used to estimate salinity values for temperature-only instruments at each mooring location^35^. We validated the fit by comparing estimated salinity with with-held mooring voyage CTD salinity profiles. The root-mean-square-errors for each mooring were 0.053 (EAC0500), 0.044 (EAC2000), 0.041 (EAC3200), 0.038 (EAC4200), 0.038 (EAC4700) and 0.040 (EAC4800).

Sloyan *et al*.^8^ undertook an extensive validation of the ITCOMPSOM method that was used to fill data gaps in the mooring data. This validation included two separate methods: 20% random withholding of good quality data over the entire data period and; withholding of a consecutive time and depth data for a period of 100 days and over a profile depth of 640 m between 60 m and 700 m of fully known good quality data. These two data validation methods simulate random loss of data from the quality control procedure, and failure of an instrument during a mooring deployment, respectively. For 20% random withholding of fully known data, validation statistics of the *u*- and *v*-velocity components were R^2^ coefficients of 0.70 and 0.88 and root-mean-square errors of 0.038 and 0.064 m s^1^, respectively, and for salinity and temperature data, root-mean-square errors of 0.04 and 0.388 °*C*, respectively. Withholding of consecutive daily depth velocity profiles had a mean profile residual differences between true and predicted *u* and *v* velocity of 0.009 and 0.02 m s^1^, respectively. The ITCOMPSOM validation statistics were significantly better than that for data filled using a least squares regression method^8^.

## Usage Notes

The East Australian Current mooring-based gridded products^12,13,21^ provide, for the first time, comprehensive and consistent data products of an approximately 10-year time-series for the complete East Australian Current from the Australian coast, across the EAC-jet to the off-shore recirculation region. Here we provide a few examples of the potential use of the data products.

**Fig. 2:**
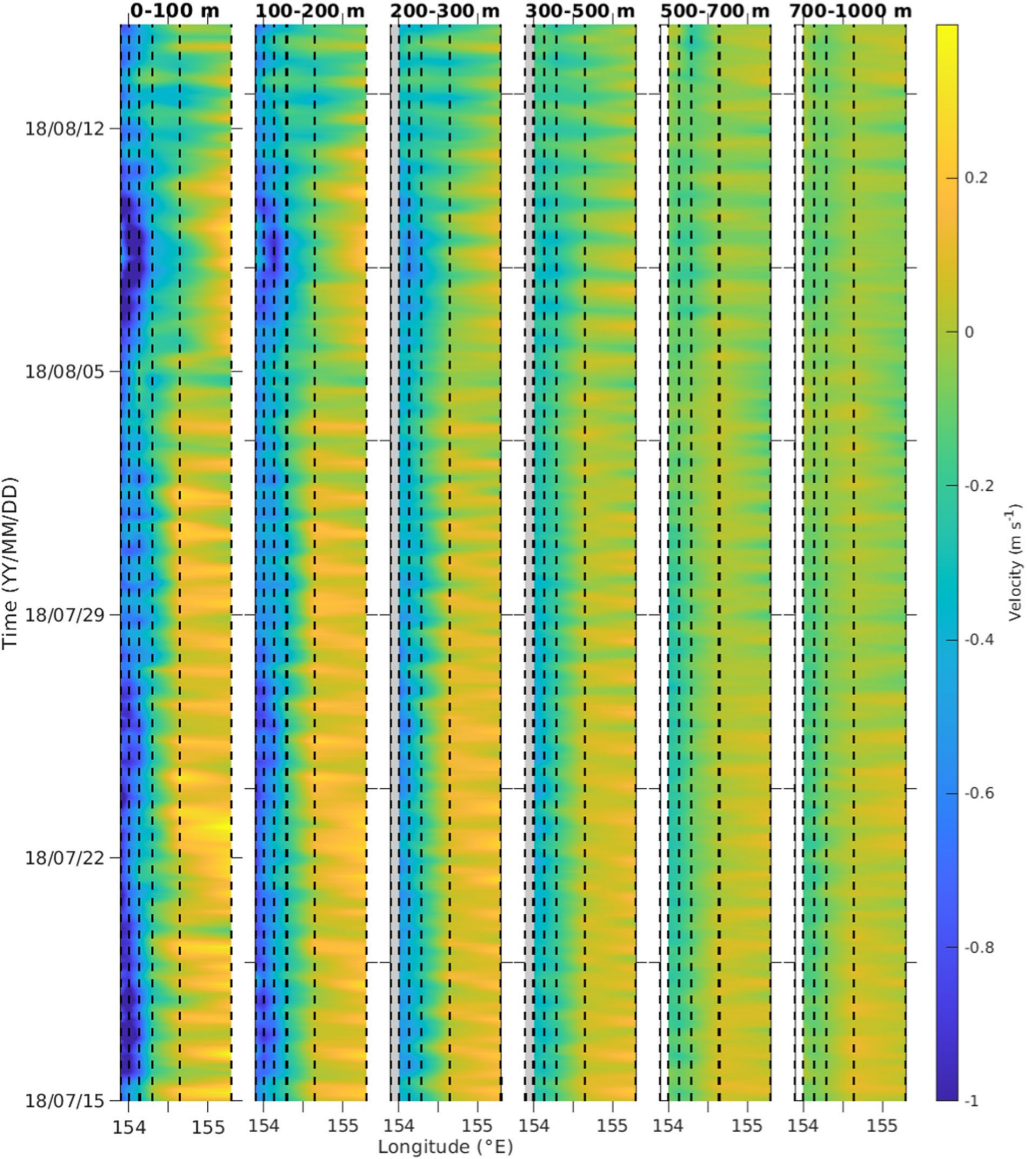
Hourly-depth EAC mooring v-velocity data from mid-July 2018 to mid-August 2018. At 27°S the inertial period is 26-hours which is close to the diurnal 24-hour tidal cycle. Variability of the v-velocity is observed corresponding to the inertial-tidal frequency. The black dashed lines show the position of the EAC moorings.

The EAC mooring hourly-depth gridded data can be used to assess the high-frequency variability of the velocity, temperature and salinity data. At 27°S the inertial period is 26-hours which is close to the diurnal 24-hour tidal cycle. Ocean current variability at the 24–26 hour period is seen in the v-velocity component at the EAC moorings (Fig. 2). Close inspection also shows variability at the semi-diurnal period (12 hours). The 1-hour time-series data can be used to investigate the time variability of the internal wave continuum and the interaction with the EAC jet current^36^.

**Fig. 3:**
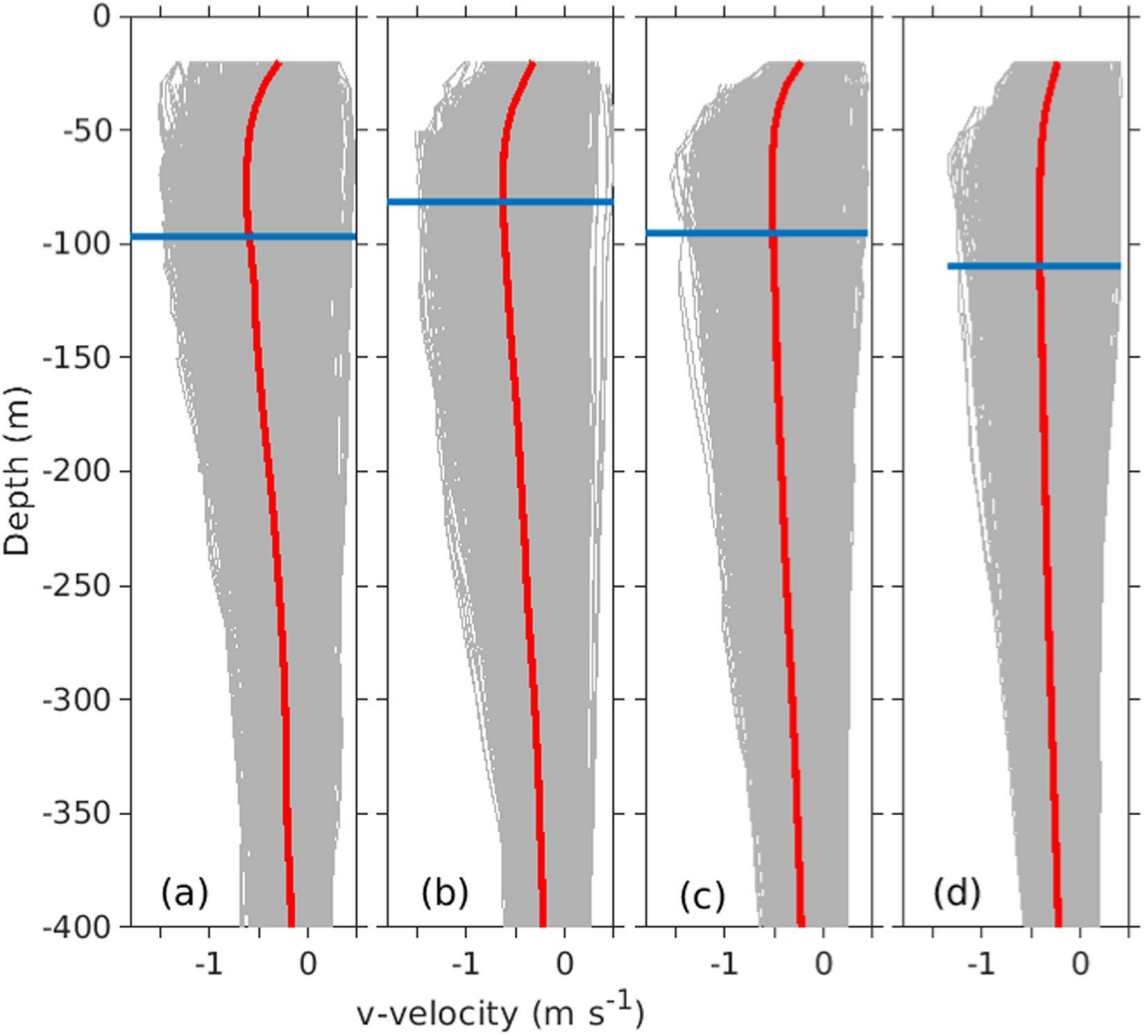
Mean v-velocity (m s^−1^) profiles for (**a**) EAC0500, (**b**) EAC2000, (**c**) EAC3200, and (**d**) EAC4200) red bold line. Grey lines shown the daily v-velocity profiles for each mooring. The mean depth of the v-velocity subsurface maximum (blue line) is shown for each mooring. The mean depth and percent time (in parentheses) a subsurface maximum is found in the time series are 96 m (58%) for EAC0500, 81 m (68%) for EAC2000, 95 m (64%) for EAC3200 and 110 m (78%) for EAC4200.

The 5-day filtering removes the high-frequency signals from the daily-depth gridded data product. Thus the daily-depth gridded product can by used to investigate the low-frequency variability at the individual moorings using either the non-filled or SOM filled time-series data. Previous studies^11^, have shown that the EAC jet has a subsurface maximum that wobbles in an east-west direction over the continental slope. Analysis of the unfilled v-velocity time-series at the EAC0500, EAC2000, EAC3200 and EAC4200 mooring show the considerable spread of v-velocity magnitude between 0–400 m (Fig. 3, grey lines). From the v-velocity profiles the time mean velocity (bold red line) and the mean depth of the subsurface velocity maximum (horizontal bold blue line) can be calculated. The v-velocity profiles show that the subsurface velocity maximum is found at a similar depth at the EAC0500 and EAC2000 moorings of 85 m and 82 m, respectively, and deepens further offshore to 95 m at EAC3200 and 110 m at EAC4200. From the v-velocity profiles we also find that the subsurface velocity maximum is found on 57%, 68%, 64% and 78% of the time at the EAC0500, EAC2000, EAC3200 and EAC4200 moorings, respectively. The daily-depth gridded product can be used to investigate boundary current dynamics including the influence of basin scale forcing and shelf forcing on EAC characteristics such as the subsurface velocity maximum and more.

The EAC depth, distance and time mooring product provides a continuous time series of EAC velocity, temperature and salinity at a 1–2 km resolution along the mooring line. Here the u- and v-velocity components are combined into the complex velocity vector, *U* = *u* + i*v. *U* is rotated −20° from true north to orientate the velocity along- and across-the primary axis of the continental slope (Fig. 1). We use the rotated velocity vector data in this analysis. Using the Gibbs Seawater oceanographic toolbox of TEOS-10^25^ we calculate pressure, absolute salinity, and conservative temperature. We use eos80_legacy_gamma_n software to calculate neutral density. TEOS-10 and legacy neutral density code were downloaded from https:https://www.teos-10.org.

**Fig. 4:**
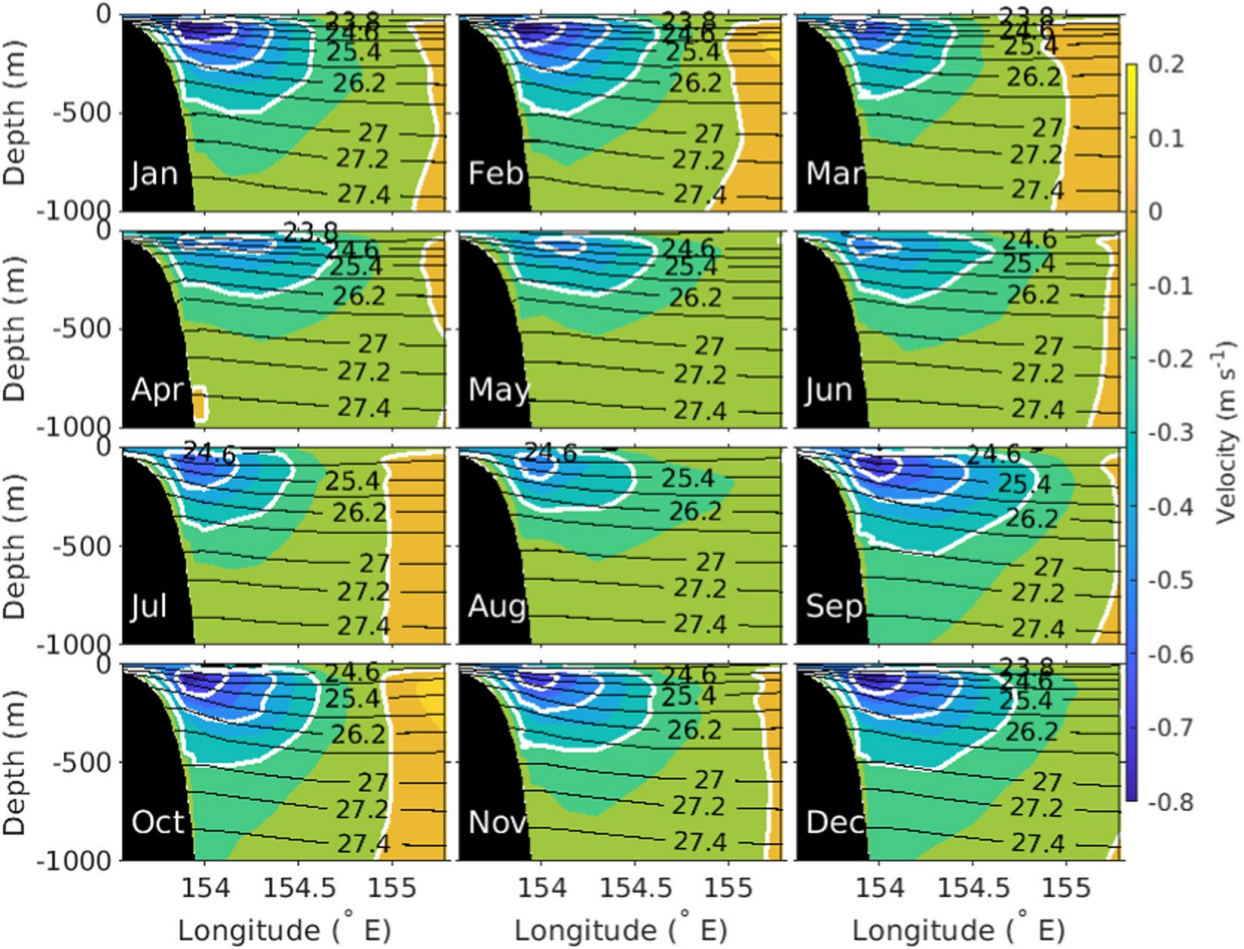
Annual seasonal cycle of along-slope velocity (m s^−1^, color) with neutral density (*γ*^*n*^, kg m^−3^, black contour lines) also shown. Along-slope velocity contours of −0.6,−0.4, −0.2 and 0 m s^−1^ are colored white.

**Fig. 5:**
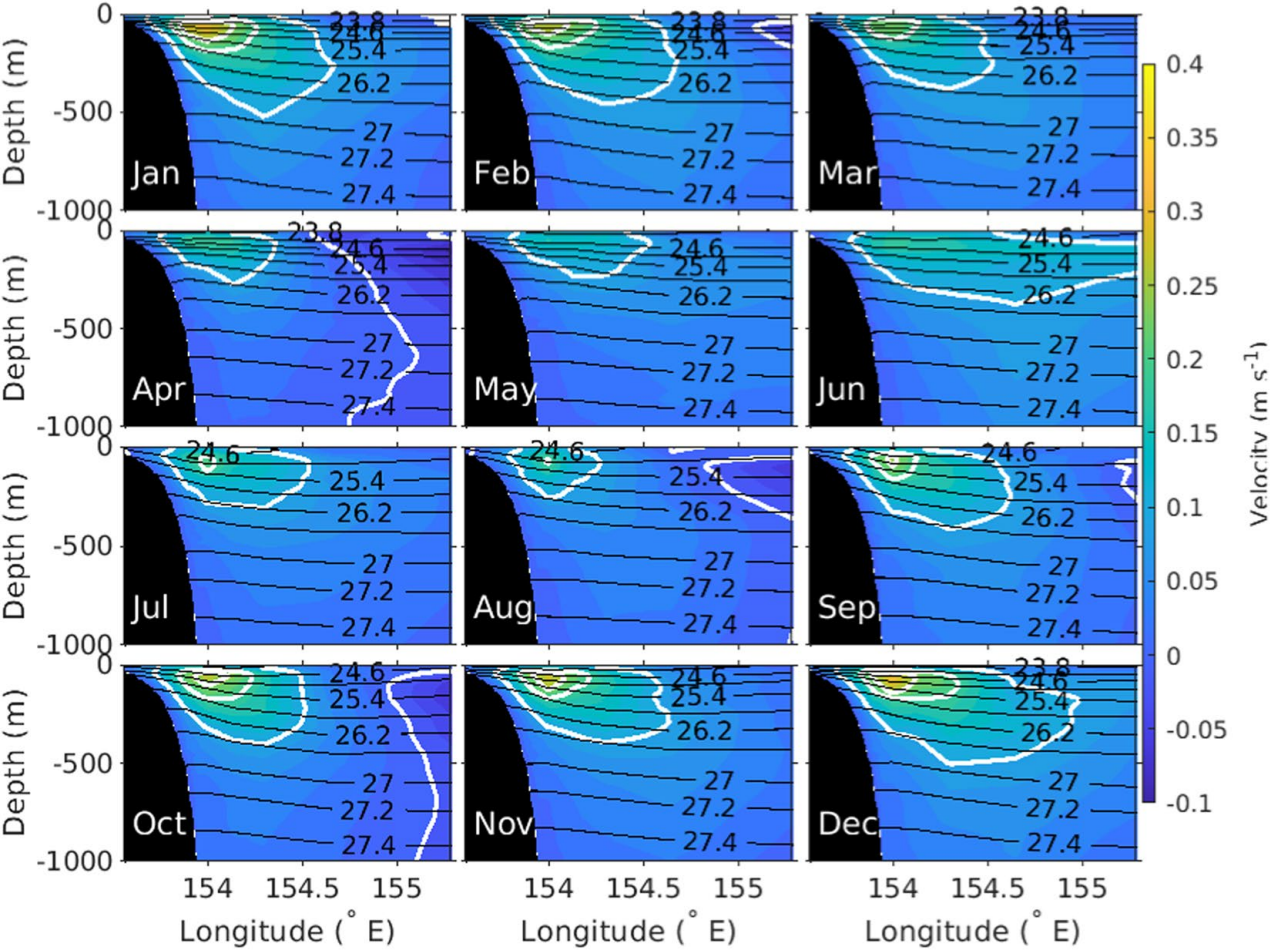
As for Fig. 4 but for across-slope velocity. Across-slope velocity contours of 0, 0.1, 0.2 and 0.25 m s^−1^ are colored white.

**Fig. 6:**
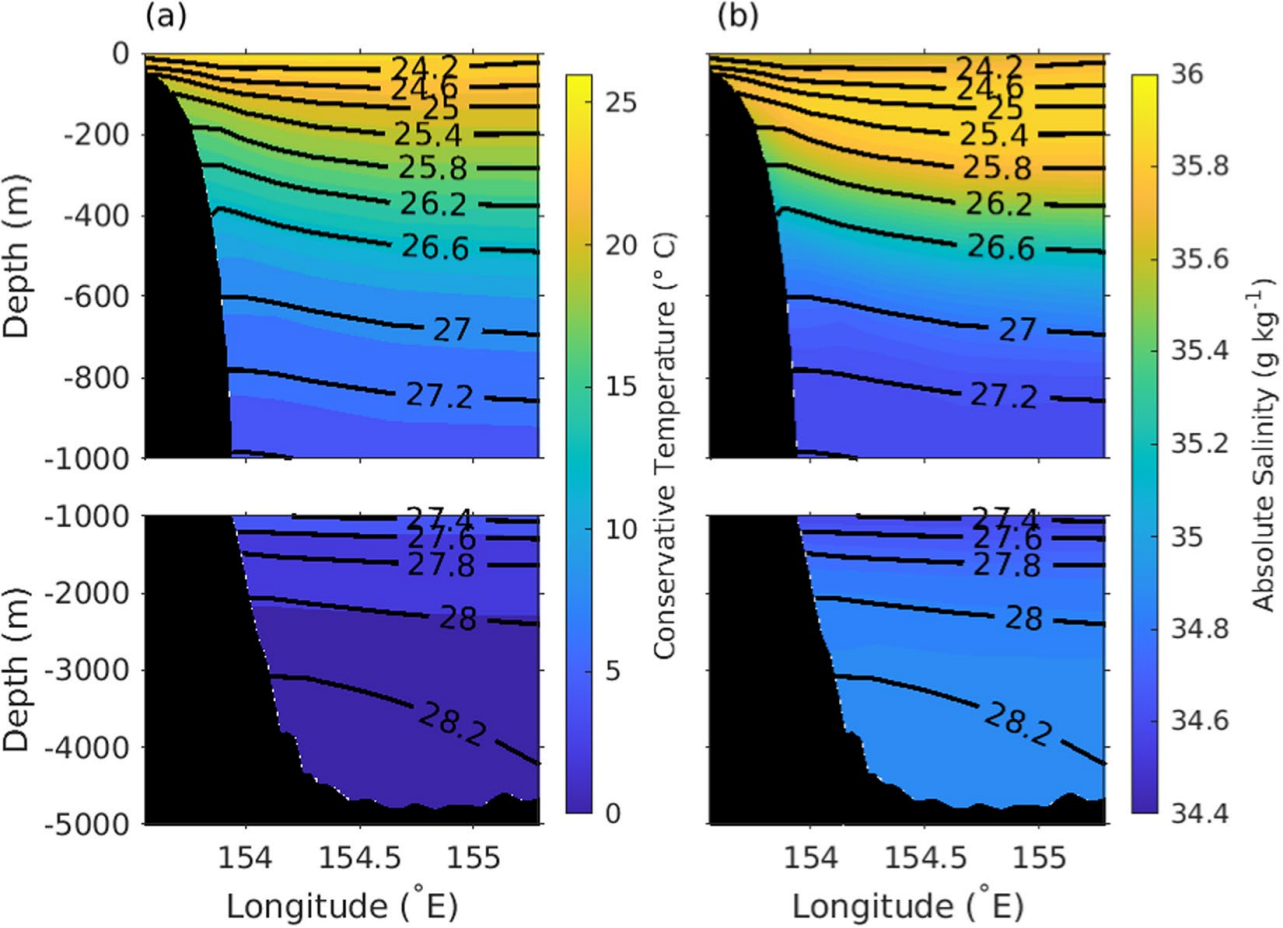
2012–2022 mean vertical distribution of (**a**) conservative temperature (°C) and (**b**) absolute salinity (g kg^−1^) along the mooring line. Neutral density (*γ*^*n*^, kg m^−3^, black contour lines) are also shown.

**Fig. 7:**
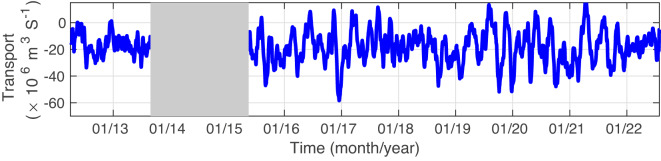
Time-series of along-slope EAC volume transport (m^3^ s^−1^, 1 Sv = 1×10^6^ m^3^ s^−1^) between 0 to 1500 m. The period between September 2012 and April 2015 when the EAC mooring array was not deployed is shown in grey.

The EAC along- and across-slope velocity time-series data are used to determine the annual cycle (Figs. 4, 5). The annual cycle of along-slope and across-slope velocity shows that a strong EAC jet is found on the continental slope from September to February, and the EAC jet weakens and moves slightly off-shore between March to August. The time mean EAC conservative temperature and absolute salinity shows the mean vertical property distribution (Fig. 6). The highest mean conservative temperature in the upper 1000 m is centred around 154°E coincident with seasonal location of the jet (Figs. 4, 5). The mean absolute salinity has a complex vertical and spatial structure with a surface minimum, subsurface maximum centered at 100 m and a subsurface minimum between 800 m to 1000 m. The surface salinity minimum is most prominent over the continental slope, and the surface salinity maximum and minimum extend westward from the eastern edge of the mooring array. Using the depth, distance and time gridded product we have calculated the EAC transport (0–1500 m) time series across the mooring array (Fig. 7). The EAC transport is highly variable with a maximum southward transport of approximately −60 Sv. There are periods where the net EAC transport is northwards, with maximum northward transport of 20 Sv. From the transport time series a mean net transport across the entire EAC of −18.15 ± 12.04 Sv is found. This is similar to previous estimates; −22.1 ± 7.5 Sv (0–2000 m) for the first 18-month period of the mooring array^11^ and −17.0 ± 2.5 Sv from a combined 10-year time-series using high-resolution expendable bathythermograph (HR-XBT) data and Argo data^37^. However, the standard deviation of the mean transport is significantly larger in the EAC mooring product compared to previous estimates.

The EAC mooring gridded products provide, for the first time, direct observational-based estimates of the EAC velocity temperature and salinity over the entire water column. Using the data products analysis of the temporal and spatial variability of the EAC currents, temperature and salinity will provide improved insights into the variability of the EAC and combined with other observational and model data lead to a more complete understanding of the western boundary current dynamics.

## Data Availability

Matlab code used to create the gridded product for each mooring^12,13^ is available at https://github.com/csiro-oceandata-code/IMOSMooringGridding/. The SOM code used to create the SOM-filled dataset is available from https://github.com/ilarinieminen/SOM-Toolboxandhttps://github.com/ilarinieminen/SOM-Toolbox or http://www.cis.hut.fi/projects/somtoolbox/http://www.cis.hut.fi/projects/somtoolbox/. Matlab function griddata using natural neighbor interpolation method was used to create the time, distance and depth gridded data product.
